# Seroprevalence of HTLV infection in Spain among immigrant pregnant women

**DOI:** 10.1186/1742-4690-8-S1-A87

**Published:** 2011-06-06

**Authors:** Ana Treviño, Rafael Benito, Estrella Caballero, Jose Manuel Ramos, Lourdes Roc, Jose María Eiros, Antonio Aguilera, Juan García, Carmen Cifuentes, Goitzane Marcaida, Carmen Rodríguez, Matilde Trigo, Luis Arroyo, Carmen de Mendoza, Vincent Soriano

**Affiliations:** 1 Hospital Carlos III, Madrid, Spain; 2 Hospital Clínico Universitario Lozano Blesa, Zaragoza, Spain; 3 Hospital Vall dÂ´Hebron, Barcelona, Spain; 4 Hospital General, Elche, Spain; 5 Hospital Miguel Servet, Zaragoza, Spain; 6 Hospital Clínico Universitario, Valladolid, Spain; 7 Hospital Conxo, Santiago de Compostela, Spain; 8 Hospital Cristal-Piñor, Orense, Spain; 9 Hospital Son Llàtzer, Palma de Mallorca, Spain; 10 Hospital General Universitario, Valencia, Spain; 11 Centro Sanitario Sandoval, Madrid, Spain; 12 Complejo Hospitalario, Pontevedra, Spain; 13 Hospital Universitario de Canarias, Santa Cruz de Tenerife, Spain

## Background

The overall seroprevalence of HTLV infection among pregnant women in Spain is below 0.02% and therefore universal screening is not recommended. However, given that a large proportion of immigrant pregnant women come from HTLV-1 endemic regions, this population might warrant specific screening considerations.

## Methods

From January 2009 to December 2010, a cross-sectional study was carried out among immigrant pregnant women attended at 14 different Spanish hospitals. An enzyme immunoassay (EIA) was used for testing serum HTLV-1/2 antibodies, being reactive samples further confirmed by Western blot.

## Results

A total of 3,337 pregnant women were examined. The origin was as follows: Latin America 1579 (47%), East Europe 606 (18%), North Africa 507 (16%), Sub-Saharan Africa 316 (9%), Western Europe 116 (5%), and Asia 163 (5%). A total of 7 samples were confirmed as HTLV positive, 6 HTLV-1 and 1 HTLV-2. HTLV-1 was found in 5 women coming from Latin America and 1 from Morocco. The only single HTLV-2 woman came from Ghana.

The overall HTLV seroprevalence in immigrant pregnant women was 0.2%, being 0.3% among Latin Americans and 0.2% among Africans. It was absent among women coming from other regions.

## Conclusions

The seroprevalence of HTLV infection among immigrant pregnant women in Spain is 0.2%, being all cases found among women from Latin America and Africa. Given the benefit of preventing vertical transmission of HTLV, antenatal HTLV screening could be advisable in pregnant women coming from these regions.

